# Unpacking gender wage inequality in poultry farming: evidence from Togo through Oaxaca-Blinder analysis

**DOI:** 10.3389/fsoc.2026.1788674

**Published:** 2026-06-29

**Authors:** Atsu Frank Yayra Ihou, Diarra Harouna, Korem Ayira, J. Paul Mansingh, Sanogo Adama

**Affiliations:** 1 Department of Agricultural Extension and Economics, VIT School of Agricultural Innovations and Advanced Learning (VAIAL), Vellore Institute of Technology (VIT), Vellore, Tamil Nadu, India; 2 Marketing and Socio-Economics of Poultry Farming, Regional Excellence Center for Avian Sciences, University of Lomé (CERSA-UL), Lomé, Togo; 3 Department of Economics, Faculty of Economics and Management, University of Lomé, Lomé, Togo

**Keywords:** gender, Oaxaca-Blinder decomposition, poultry industry, wage discrimination, wage inequality

## Abstract

This study investigates gender wage inequality in poultry industries, a relevant issue in the ongoing debates on workplace discrimination. The study used the Oaxaca-Blinder decomposition method to assess the portion of wage differences explained and unexplained. The methodology involves the collection of primary data from 200 employees across six prefectures in the Maritime region of Togo. The analysis reveals that 35.10% of the total wage gap is explained by differences in individual characteristics between genders, such as age, experience, and level of responsibility, while 64.90% of the gap remains unexplained. This unexplained portion is likely due to wage discrimination. These findings strengthen the hypothesis that wage discrimination is a major factor contributing to the observed wage gap. This study suggests that policies should still be combating this discrimination which is crucial for ensuring greater equity in the poultry sector and, more broadly, in other economic sectors.

## 1 Introduction

Gender inequality continues to be one of the most pressing challenges in the global workforce, with women systematically facing disadvantages in access to economic opportunities, employment, and remuneration. This issue transcends geographical boundaries, impacting not only the developed world but also emerging and developing economies. The gender wage gap, in particular, has remained a crucial focus for research and policy development as it has direct implications for economic growth, development, and the empowerment of women. Addressing gender inequality is not only a matter of social justice but also a key factor in achieving sustainable and inclusive economic progress. Women worldwide, despite their significant contributions, earn considerably less than men, with the gender pay gap globally ranging from 15% to 30%. In regions like South Asia, the Middle East, and North Africa, these disparities are especially pronounced, with women facing substantial barriers in entering the workforce and achieving economic parity (Clark, 2012). In Sub-Saharan Africa, despite the dynamic role women play in economic activities, gender disparities persist, with women facing lower wages, greater obstacles in entrepreneurship, and limited access to resources like land and credit (Georges-Kot, 2020; African Development Bank, 2016).

In Sub-Saharan Africa, the wage gap between men and women is particularly stark. The International Labor Organization (ILO) reports that men in this region earn more than twice as much as women in median monthly income. Furthermore, a 2022 World Bank study on female entrepreneurship in Africa found that women entrepreneurs, on average, earn only two-thirds of the income generated by their male counterparts. These income disparities have far-reaching implications, not just for women's financial independence but also for the broader economic development of the region. Gender inequality in Sub-Saharan Africa costs the continent approximately $95 billion annually, a figure that represents about 6% of its GDP (UNDP, 2016). One of the key sectors where gender inequality is most visible is agriculture, which remains central to the economic structure of many African countries. Women make significant contributions to agriculture, working in both formal and informal roles, yet they often face considerable barriers in terms of access to land, technology, and finance, which restrict their economic opportunities. This disparity in the agricultural sector has profound consequences not only for individual women but for national economies as a whole.

Despite extensive research on gender inequalities in various sectors, there is a noticeable gap in studies examining gender disparities within the poultry industry in West Africa, particularly in Togo. Although Togo has recently become a leader in poultry science in the region, contributing to both the growth of its economy and the agricultural industry, there is little research addressing the gender wage gap within this sector. This presents an important opportunity to explore gender inequality in Togo's rapidly growing poultry industry and assess the extent of these disparities. Previous studies on gender inequality in other sectors reveal valuable insights into the broader dynamics of gender-based disparities in the workplace. For instance, research consistently shows that women are paid less, have fewer opportunities for career advancement, and face greater barriers to entrepreneurship compared to their male counterparts (Adair and Gherbi, 2022; Georges-Kot, 2020). These findings are consistent with global and regional reports, such as the World Bank's 2022 report on female entrepreneurship and the UNDP's analysis of the economic cost of gender inequality in Sub-Saharan Africa (UNDP, 2016; Programme d'Appui au Développement de l'Élevage, 2021). These studies highlight socio-cultural factors, such as gender norms, limited access to education and training, and discriminatory practices, which perpetuate gender inequality in the workforce.

Togo, particularly the Maritime Region, is home to about 70% of the country's poultry production, making this area a key site for the development of the poultry industry. Despite the significant presence of women in agriculture, gender inequalities persist in the region, especially in terms of income disparity and access to resources. According to the 2022 general population census, women make up 51.3% of the total population in Togo, and 51.21% of the rural population. Approximately 54% of the active workforce is employed in agriculture, with women accounting for 53.46% of those employed in this sector. Despite their significant contributions, women in the agricultural sector, including poultry farming, earn only a fraction of the income generated by their labor compared to their male counterparts (Ministry of Agriculture, Togo). The persistent wage disparity reflects the broader economic and social inequalities women face in Togo's labor market, particularly in the rural regions that drive the agricultural economy.

The aim of this paper is to examine gender-based wage disparities in the poultry farming sector of the Maritime Region of Togo. Specifically, this study aims to analyze the wage differences between men and women in this industry, focusing on the factors that contribute to the persistence of these inequalities. This research will fill a significant gap in the literature on gender inequality in the poultry industry, providing evidence-based recommendations for policy-makers, business leaders, and agricultural stakeholders to promote gender equality in this key sector. Given the rapid expansion of Togo's poultry industry, it is crucial to ensure that women equally benefit from the economic opportunities it provides. This research aims to contribute to the development of policies and practices that foster gender equality in Togo's poultry industry and beyond, offering insights into how gender parity can be achieved in agriculture more broadly.

### 1.1 Theoretical background

In the literature, the topic generally draws on at least two theories: the human capital theory by Mincer and Polachek (1974) and Polachek (1981), and the theory of labor market discrimination by Becker (1971) and Bergmann (1974). The discrimination theory constitutes the main rival hypothesis to orthodox human capital analysis. It posits that gender wage differences result from discrimination exerted by firms on the labor market, where women are offered lower wages. Bergmann develops another theory of wage discrimination, suggesting that the exclusion of women from the male-dominated segments of the labor market leads to an oversupply of labor in sectors primarily occupied by women, creating downward pressure on wages in these segments. This feedback theory traces back to Blau.

According to Becker, the discrimination theory introduces the impact of the environment on individual economic outcomes, challenging the assumption that agents optimize their economic decisions flexibly. One of Becker's conclusions is that labor market discrimination occurs when two individuals with identical productive characteristics receive different wages or do not have access to comparable jobs in terms of prestige and expected compensation.

Polachek argues that differences in labor market participation and mobility rates between men and women can lead to disparities in human capital investment (such as education level or professional experience), thus resulting in productivity differentials. Women tend to specialize in occupations where career interruptions do not penalize them, namely in low-skilled jobs.

## 2 Methodology

### 2.1 Study area

To ensure that the sample is as representative as possible, the study used a two-stage stratified sampling method. The Maritime Region, which is divided into eight districts (Golfe, Agoè-nyivé, Lacs, Bas-Mono, Vo, Yoto, Zio, and Avé), was considered based on data from the National Association of Poultry Professionals of Togo (ANPAT, 2024). Six districts were selected namely, the districts of Avé, Golfe, Lacs, Vo, Agoè-nyivé, and Zio due to the high concentration of poultry farmers in these areas.

### 2.2 Sampling design

For this study, primary data was collected from 200 poultry farms in the Maritime Region of Togo. To ensure that the sample is as representative as possible, the study used a two-stage stratified sampling method. In the first stage, the Maritime Region, which is divided into eight districts, was considered based on data from the National Association of Poultry Professionals of Togo (ANPAT, 2024). Six districts were selected due to the high concentration of poultry farmers in these areas. In the second stage, 200 poultry farms from the study area were surveyed randomly.

The sample size was determined using the formula from Franklin et al. (2010), see Table 1.


$$\begin{array}{l} n_{1}=\frac{Z^{2}\hat{P}\left(1-\hat{P}\;\right)}{e} \end{array}$$

with the following parameters:

**Table 1:** Determination of sample size in the study area.

Symbol	Name	Value
***N***	**The poultry unit population size**	**302**
E	The margin of error	5%
Z	95% confidence level	1.96
P	The percentage of companies exhibiting the observed characteristic	50%
***n*_2_**	**Sample size**	**169**

Source: Author, 2024.

The confidence interval is 95%

Z = a value corresponding to the desired confidence level

$\hat{P}\;=$ the estimated proportion precision

e = margin of error

*n* = initial sample size


$$\begin{array}{l} n=\frac{{\left(1,96\right)}^{2}\left(0,5\right)\left(0,5\right)}{{\left(0,05\right)}^{2}}=384 \end{array}$$

With this formula, the adjusted sample (*n*_2_) size that accounts for the population size:


$$\begin{array}{l} n_{2}=n_{1}\frac{N}{N+n_{1}}=384\frac{302}{\left(302+384\right)}=169 \end{array}$$

This yielded a sample of 169 poultry farms based on data from ANPAT (2024).

### 2.3 Econometric analysis model

The Oaxaca-Blinder decomposition method (Oaxaca, 1973; Blinder, 1973) is widely used for measuring wage discrimination. It allows the wage gap between groups to be partitioned into two components: one explained by observable characteristics (such as education, experience, etc.) and the other attributed to differences in characteristics based on gender, which is interpreted as discrimination (see Table 2). The objective is to evaluate the extent of gender wage inequality that cannot be explained by productive characteristics of individuals.

**Table 2:** List of retained variables.

Variable dependent	Modalities/measures
The wage	Numeric
Variables independents	Modalities/measures
Gender	0 = Male and 1 = Female
Education level	1 = illiterate, 2 = Primary, 3 = Secondary, 4 = University graduate.
Experience	Numeric
Age	1 = Below 25; 2 = 26 to 35; 3 = 36 to 45; 4 = 46 to 55 and 5 = More than 55 years
Marital status	1 = Single; 2 = Married; 3 = Divorced and 4 = Widowed
Employee status	1 = Executive 0 = Worker
Religion practiced	1 = Christian; 2 = Muslim and 3 = Animist

Source: Author, 2024.

#### 2.3.1 Specifications of the Oaxaca-Blinder linear model

Given that the wage formation process for men and women is assumed to differ, the equations for the two groups must be estimated separately. These can be written as following Equations 1–4:


$$\begin{array}{l} lnW_{i}^{m}=\propto^{m}+\beta^{m}X+\in_{i}^{m} \end{array}$$ (1)

$$\begin{array}{l} lnW_{i}^{f}=\propto^{f}+\beta^{f}X+\in_{i}^{f} \end{array}$$ (2)

Where:

*lnW* = natural logarithm of monthly wage

*X* = vector of explanatory variables

β = vector of coefficients of the explanatory variables

ϵ = residual term, to be estimated by OLS (Ordinary Least Squares)

Since the OLS regression line passes through the mean point, the two initial equations can be rewritten as:


$$\begin{array}{l} lnW_{i}^{m}=\propto^{m}+\beta^{m}X \end{array}$$ (3)

$$\begin{array}{l} lnW_{i}^{f}=\propto^{f}+\beta^{f}X \end{array}$$ (4)

The total wage difference between men and women can be written as:


$$\begin{array}{l} lnW_{i}^{m}-lnW_{i}^{f}=\propto^{m}-\propto^{f}+\beta^{m}X^{m}-\beta^{f}X^{f} \end{array}$$ (5)

The transformations of Equation 5 lead to:


$$\begin{array}{l} lnW_{i}^{m}-lnW_{i}^{f}=\propto^{m}-\propto^{f}+{\left(\beta^{m}-\beta^{f}\right)}^{*}X^{f}+\beta^{m^{*}}\left(X^{m}-X^{f}\right) \end{array}$$ (6)

$$\begin{array}{l} lnW_{i}^{m}-lnW_{i}^{f}=\propto^{m}-\propto^{f}+{\left(\beta^{m}-\beta^{f}\right)}^{*}X^{m}+\beta^{f*}\left(X^{m}-X^{f}\right) \end{array}$$ (7)

Where:

∝^*m*^ − ∝^*f*^ + (β^*m*^ − β^*f*^)*X^*f*^ = explained component

β^m*^(*X*^*m*^ − *X*^*f*^) = discriminatory component

Equation 6 measures wage discrimination at the level of women, while Equation 7 does so for men. The equation for men (Equation 7) implicitly assumes that the wage equation for men represents the “normal” wage rate that the labor market should grant to individuals possessing certain productive characteristics.

As the dependent variable is specified in natural logarithm, estimated coefficients are transformed using (*e*^β^−1) × 100 to obtain the percentage change in wages associated with a one-unit change in the explanatory variables.

The analysis was performed using STATA 17.

## 3 Results and discussions

### 3.1 Descriptive and inference statistics

The analysis of Table 3 presents the average salaries by gender across the sample, which includes 136 males and 64 females. It shows that the average salary for men is 72,536.76 CFA, compared to 53,046.87 CFA for women. The average salary within the poultry farms of the Maritime Region of Togo is 66,300.00 CFA. This indicates that men have a higher average salary than women.

**Table 3:** Descriptive statistics of average salaries by gender in the study area in CFA.

Genre	Minimum	Mean	Maximum	Std Dv	*N*
Male	30,000.000	72,536.76471	225,000.000	37,659.519103	136
Female	25,000.000	53,046.87500	100,000.000	24,387.206956	64
Total	25,000.000	66,300.00000	225,000.000	35,120.896727	200

Source: Author, 2024.

#### 3.1.1 Multicollinearity test outcome

The Variance Inflation Factor (VIF) results in Tables 4, 5 indicate that multicollinearity is not a concern in the model, as all VIF values are well below the commonly accepted threshold of 10. The mean VIF of 1.47 further confirms a low level of correlation among explanatory variables. Therefore, the estimated coefficients can be considered stable and reliable.

**Table 4:** The multicollinearity test.

Variable	VIF	1/VIF
Education	1.92	0.520211
Experience	1.71	0.585201
Age	1.68	0.596008
Marital status	1.50	0.666407
Status (cadre)	1.47	0.678429
Exploitation size	1.34	0.747878
Gender	1.08	0.925436
Religion	1.06	0.939540
**Mean VIF**	**1.47**	—

**Table 5:** OLS estimation of the monthly salary expressed in natural logarithm.

Variables	(1)	%ΔY	(2)	%ΔY	(3)	%ΔY
	Together		Man		Woman	
Gender	–0.255^***^	−22.5				
	(0.0318)					
Age	**0.173** ^**^	**18.9**	**0.190** ^**^	20.9	0.108	11.4
	(0.0736)		(0.0932)		(0.0919)	
Age^2^	−0.0123	−1.22	−0.0124	−1.23	−0.0167	−1.66
	(0.0138)		(0.0160)		(0.0168)	
Experience	0.117	**12.4**	**0.267** ^*^	30.6	0.0764	7.9
	(0.122)		(0.148)		(0.225)	
Exp	−0.00941	−0.94	−0.0307	−3.02	−0.00707	−0.7
	(0.0339)		(0.0382)		(0.0657)	
Status marital (Married)	−0.0468	−4.58	0.0182	**1.84**	–0.248^***^	–**21.9**
	(0.0351)		(0.0354)		(0.0839)	
Status marital (Widowed)	**−0.210** ^*^	−18.9	−0.107	−10.1	−0.168	−15.5
	(0.107)		(0.118)		(0.140)	
Status marital (Divorced)	0.0554	5.69	0.00309	0.31	−0.0311	−3.06
	(0.0761)		(0.0627)		(0.0819)	
Education (Primary)	0.0226	2.29	−0.0257	−2.54	0.0734	7.61
	(0.0474)		(0.0620)		(0.0573)	
Education (Secondary)	**0.290** ^***^	**33.6**	**0.196** ^***^	**21.7**	**0.379** ^***^	**46.1**
	(0.0554)		(0.0639)		(0.0763)	
Education (High school)	**0.609** ^***^	**83.8**	**0.479** ^***^	**61.5**	**0.660** ^***^	**93.5**
	(0.0736)		(0.0781)		(0.105)	
Religion (Muslim)	**−0.0909** ^**^	–**8.69**	**0.0784** ^*^	**8.15**	–0.162^**^	–**14.9**
	(0.0424)		(0.0401)		(0.0628)	
Religion (Animist)	0.00810	0.81	0.0337	3.43	−0.0672	−6.5
	(0.0495)		(0.0688)		(0.0492)	
Status (cadre)	**0.208** ^***^	**23.1**	**0.194** ^***^	21.4	0.0185	1.87
	(0.0498)		(0.0490)		(0.0807)	
Exploitation size	**0.165** ^***^	**17.9**	**0.0743** ^*^	**7.71**	**0.155** ^***^	**16.8**
	(0.0336)		(0.0411)		(0.0462)	
Cons	**10.04** ^***^		**10.00** ^***^		**10.22** ^***^	
	(0.127)		(0.148)		(0.239)	
Observations	200		136		64	
*R*–squared	0.858		0.869		0.938	

1%^***^, 5%^**^ and 10%^*^.

#### 3.1.2 Heteroskedasticity test outcome

Breusch–Pagan/Cook–Weisberg test for heteroskedasticity

Assumption: Normal error terms

Variable: Fitted values of Salaires

H0: Constant variance

chi2(1) = 63.73

Prob > chi2 = 0.0000

The Breusch–Pagan test indicates the presence of heteroskedasticity. To address this issue, the Oaxaca–Blinder decomposition was estimated using robust standard errors [pooled vce (robust)] to ensure consistent statistical inference.

### 3.2 Econometric analysis

As it is demonstrated in Table 6, there are a number of salient determinants of wage inequality, among which gender has proven to be one of the most persistent and salient determinants. The value of the coefficient that deals with women is minus and is significant at the 1% level, which means that women are about 22.5% less paid when compared to their male counterparts. This result agrees with previous research by Johnson (2016) and Gouider (2009), who reported similar gender wage gaps in Togo and Tunisia despite adjustments on education, experience, and a multitude of socioeconomic covariates. The persistence of this difference implies that women are still disadvantaged in the labor market due to structural and institutional processes that are not observable on the surface.

**Table 6:** Presentation of the Oaxaca-Blinder decomposition of monthly salary.

	Average wage difference	%ΔW			
Average salary of men	11.083^***^		(284.19)		
Average salary of women	10.780^***^		(193.34)		
Average gender wage gap	**0.302** ^***^	**35.2**	(4.44)		
Overall decomposition	Part explained		Part unexplained		interaction
	0.106^*^		0.289^***^		−0.093^**^
	(1.75) 35,10%		(7.35) 64,90%		(−2.43)
Detailed decomposition	Part explained	**%Δ**	Part unexplained	**%Δ**	interaction
Age	−0.004 (−0.14)	−0.4	0.442 (1.16)	55.6	−0.042 (−0.98)
Age^2^	−0.001 (−0.05)	−0.1	−0.100 (−0.50)	−9.52	0.017 (0.48)
Status marital	0.12 (1.02)	12.7	0.182^*^ (1.69)	**11.9**	−0.011 (−0.95)
Education	0.046 (1.17)	4.71	−0.226^**^ (−2.11)	**−20.2**	−0.015 (−1.03)
Religion	0.019^*^ (1.71)	**1.92**	0.169^***^ (2.78)	**18.4**	−0.029^**^ (−1.86)
Experience	−0.001 (−0.13)	−0.1	0.505 (1.14)	65.7	−0.014 (−0.43)
Statu in the entreprise	0.005 (0.72)	0.5	0.204^**^ (2.02)	**22.6**	0.012 (0.99)
Exploitation size	0.030^*^ (1.72)	**3.05**	−0.161 (−1.61)	−14.9	−0.014 (−1.22)
_cons			−0.599^*^		
	(−1.92)		
*N*	200.000				

1%^***^, 5%^**^ and 10%^*^.

There is also a strong effect of age on earnings especially among men. An annual increase of one year in age corresponds to a salary increase of about 20.9% which is attributed to career advancement and accrual of job related skills with increase in age. The negative and statistically significant squared age term, however, suggests decreasing returns as one ages and so wage growth tends to slow down or even become negative at a particular age. The trend is consistent with life-cycle earnings pattern, which Etoundi Atenga et al. (2013) reported in Cameroon.

Education has a strong and positive effect on wages between the two genders, albeit in varying degrees (Mahugnon and Hamdy, 2023). The increase in salaries through secondary schooling increases by 21.7% among men and 46.1% among women, respectively; an increase in salaries with tertiary education extends to 61.5% and 93.5%, respectively. These findings are very close to the human-capital theory that is developed by Becker (1964) and Mincer and Polachek (1974), which assumes that education is the primary determinant of productivity and earnings. The increased returns to education among women could be a result of formal qualifications partially canceling labor-market discrimination by signaling competency and expertise, they are not enough to bridge the overall gender wage gap in the sample.

There are other layers of wage inequality added on by religious affiliation and marital status. The increase in salaries by Muslim men is 8.15%, and by Muslim women, 14.9%, indicating a strong effect of gender differences among members of the same religion group. This result is in line with Ekamena Ntsama (2014) and Nazari (2022) and highlights the role of labor-market outcomes and gender roles, which are affected by cultural norms. These discrepancies are also strengthened by marital status. Marriage is linked to a wage cut of 24.8 rates of female workers, an issue that has long been held by society, showing that the work of married women is undervalued, at the same time, marital status raises the earnings of men significantly, furthering the male breadwinner premium. The same trends are reported by Bera et al. (2025) and Bach et al. (2023), who suggest that marriage is often understood as an expression of stability and commitment among male groups, but as a limitation of labor supply and productivity among female groups.

Occupational roles, experience and firm-level characteristics also help to shed light on the gendered nature of wage determination. Among men, experience increases by 1%, increasing the salary by 30.6%; the returns to experience become diminishing with increased experience. The experience of women, on the other hand, is not statistically significantly reflected on the wages, implying that there must be systemic impediments limiting the ability to translate experience into earnings (Bera et al., 2025; Bach et al., 2023). The managerial or supervisory role also increases the salary of men by 21.4%, which is significant at the 1% level, but makes no difference in the case of women, which shows that the contribution of managers is also valued in a gendered manner, as Fuller and Kim (2024) note. Firm size is gender specific, with an increasing the size of poultry operation corresponding to a 7.1–16.8 percentage pay raise to men and women, respectively, which is statistically significant at the 10 and 1 percentage thresholds, respectively. Although this implies that bigger companies can provide more unified or clearer pay scales which partially benefit women, the total managerial pay disparity remains. As Theodoropoulos et al. (2022) note, the size of firms can alleviate, although not eliminate, gender pay gaps; the proportion of female managers could help reduce the difference, especially in performance-based remuneration cultures.

#### 3.2.1 The Oaxaca-Blinder decomposition of the natural logarithm of monthly salary to assess wage differences

Table 6 presents the results of the Oaxaca-Blinder decomposition of gender wage differentials. The estimated average monthly salary for men is higher than that for women. Specifically, the natural logarithm of the average estimated salary for men is 11.083, compared to 10.780 for women, reflecting a gender wage gap of 0.302.

The Oaxaca–Blinder decomposition shows that approximately 35.1% of the observed wage gap is explained by differences in observable characteristics (endowments), while 64.9% remains unexplained by the variables included in the model. The unexplained component captures differences in the returns to these characteristics between men and women and, in this sense, constitutes the primary source of the observed wage gap. This suggests that men and women with similar observable attributes are rewarded differently in the labor market.

However, following standard econometric practice, this unexplained portion should not be interpreted entirely as discrimination. It may also reflect the influence of unobserved factors, omitted variables, such as productivity-related attributes not captured in the dataset, and potential model specification or measurement errors. Therefore, while the unexplained component provides evidence consistent with the presence of gender-based disparities in remuneration, it should be interpreted as an upper-bound estimate of discrimination rather than a precise measure. Overall, the results indicate that structural differences in the valuation of characteristics play a significant role in explaining the wage gap in poultry farming in the Maritime region of Togo.

The Oaxaca-Blinder decomposition provides meaningful data on the causes of wage differences in the Maritime region of Togo, where it can be seen that a significant part of the gender wage gap may due to discriminatory processes. The results show that a substantial 35.09% of the observed wage difference can be explained by specific features that are measurable including education, experience, and occupation job titles, but there is also a significant 64.90% to be covered. This large unaccounted element is a strong suggestion of the fact that discrimination of women in relation to wage remains even when personal characteristics are held constant. These findings are consistent with other uses of the Oaxaca -Blinder approach, which also attribute a large portion of gender wage differentials to discrimination as opposed to variations in productivity related considerations (OECD, 2022; Giaimo and Lo Magno, 2014). Similar findings in Spain highlight the effect of such phenomena as glass ceiling and sticky floor which reinforce wage discrimination against women (Dueñas Fernández et al., 2015; Thiongane and Kane, 2023). However, it is critical to note that though the decomposition is quite revealing of structural differences, it might be inadequate in terms of contextual factors including family processes that also influence payoff findings (Averkamp et al., 2024; Daudin, 2005; Houanti et al., 2022).

Marital status comes out as a very strong predictor of discrimination in the unexplainable part of the wage gap. The discriminatory component of wages in poultry farms increases women who are married by 11.9% which is statistically significant at the 10% level. The results support the theoretical viewpoints offered by Cain (1986) regarding labor-market discrimination and the fact that women are often subject to negative stereotypes which diminish their productivity, and men are given more positive stereotypes. These findings indicate that marriage as a penalty applies to women but not to men, thus, supporting gendered principles of labor involvement and labor productivity. These trends also contribute to the hypothesis that discrimination is not an action that can be determined by visible features but rather by social and institutional frameworks.

Education on the other hand appears to have a moderating effect by alleviating discrimination. The unexplained term of years of schooling has a negative value, which shows that the number of years of education among women reduces discrimination by 20.2%. This implies that education can be used as a partial shield, indicating to the employers competence and productivity. Yet, this is not statistically significant in the component of the explained better than the results of Alkemade et al. (2021), who note that more educated men receive higher wages when compared to women with similar education. The given discrepancy can be associated with context-specific labor-market relationships where education can alleviate discrimination but not bring about a complete equalization of returns between the sexes.

Religion and occupation also further bring light into the structural aspect of wage inequality. Muslim men are paid 1.92% more than women who possess similar characteristics and Muslim women encounter a wage discrimination of 18.4% with this variable achieving significance of 10% of the component that is explained and 1% of the component that is unexplained. These results are reflective of the findings made by Johnson (2016) in Togo and they are indicative of the convergence between gender and religious norms in determining labor-market outcomes. These disparities are further intensified by occupational status, especially in management positions, where women are found to be affected by 22.4% more than otherwise. The high coefficients at the 5% thresholds serve to highlight the fact that the female gender in managerial roles is faced with strong wage penalties, despite adjustment of qualifications and job attributes. Similar evidence in other contexts shows that occupational segregation and structural aspects play a significant role in the explained part of the wage gap (Sarma, 2022; Edelsztein, 2023), and unobserved discriminatory factors rule the unexplained part (Putri et al., 2025; Sielska, 2023). Despite the fact that some people claim that work-life balance choice and career decision can be one of the factors that explain the pay gap, the size and meaning of the unaccounted factor indicates that discrimination is still the key factor of gender wage differences.

## 4 Conclusion

This study provides an in-depth analysis of gender-based wage disparities in the poultry farms of the Maritime Region of Togo, uncovering significant inequities through descriptive statistics, econometric modeling, and the Oaxaca-Blinder decomposition. The findings shows that the wage decomposition between men and women revealed a 30% difference, isolating an unexplained residual in remuneration. Some part of this residual, attributed to wage discrimination between men and women, highlights the extent to which the principle of “equal pay for equal work” is not respected.

The results indicate that 35.10% of the wage gap is explained, while 64.90% remains unexplained, signifying significant discrimination against women in terms of pay exist even some part of this unexplained due to some unobserved factors. This undervaluation, deemed discriminatory, is largely driven in this case by factors such as marital status (married), religion (Muslim), and occupational status (managerial role). According to the findings, these factors contribute positively to discrimination against women. However, education level plays a role in reducing discrimination against women. In other words, women with relatively higher levels of education are more likely to be fairly compensated. The econometric results also align with existing literature, including Becker's human capital theory, indicating that an individual's income is a function of their level of education.

The results emphasize the need for policy interventions aimed at reducing gender wage gaps and addressing systemic inequities in labor markets. These include promoting equitable hiring practices, enhancing access to education and training for women, and implementing policies that ensure equal pay for equal work. Moreover, targeted programs to support women in leadership roles are crucial for dismantling barriers that limit their earnings potential. Future research should investigate the Impact of Policy Measures by assessing the effectiveness of existing gender equity policies in the region, such as anti-discrimination laws and wage standardization efforts, in mitigating wage disparities. Longitudinal studies could help track the progress and identify gaps in implementation.

## Data Availability

The original contributions presented in the study are included in the article/supplementary material, further inquiries can be directed to the corresponding author/s.

## References

[B1] Adair P. Gherbi H. (2022). Gender inequalities among youth in North Africa: income gaps and informal employment. Maghreb-Machrek 250–251, 167–182. doi: 10.3917/machr.250.0167

[B2] Alkemade P. Checchi D. Cissé S. Coulibaly A. David A. Koné A. . (2021). Analysis of income inequalities in Mali. Res. Pap. 1–56.

[B3] Averkamp D. Bredemeier C. Juessen F. (2024). Decomposing gender wage gaps: a family economics perspective. Scand. J. Econom. 126, 3–37. doi: 10.1111/sjoe.12542

[B4] Bach P. Chernozhukov V. Spindler M. (2023). Heterogeneity in the US gender wage gap. J. R. Stat. Soc. 187, 209–230. doi: 10.1093/jrsssa/qnad091

[B5] Becker G. S. (1964). Human Capital. New York, NY: Columbia University Press.

[B6] Becker H. S. (1971). Sociological Work. Piscataway, NJ: Transaction Publishers.

[B7] Bera M. Dubey A. Malhotra S. (2025). Revisiting earnings differentials across socio-religious groups in the regular salaried employment in India. Indian Econom. J. 73, 751–768. doi: 10.1177/00194662231201209

[B8] Bergmann B. R. (1974). Occupational segregation, wages and profits when employers discriminate by race or sex. East. Econ. J. 1, 103–110.

[B9] Blinder A. S. (1973). Wage discrimination: reduced form and structural estimates. J. Hum. Resour. 8, 436–455. doi: 10.2307/144855

[B10] Cain G. G. (1986). The economic analysis of labor market discrimination: A survey. Handb. Labor. Econ. 1, 693–785.

[B11] Clark H. (2012). What the Human Development Report Taught Us. Development Cooperation 2011 Special Edition: 50th Anniversary, (Vol 51).

[B12] Daudin G. (2005). Trade and Prosperity: France in the 18th Century, (Vol 19). Presses Paris Sorbonne.

[B13] Dueñas Fernández D. Iglesias Fernández C. Llorente Heras R. (2015). “Descomposición del GAP salarial por género en el mercado de trabajo español,” in Aportaciones a la Investigación Sobre Mujeres y Género: V Congreso Universitario Internacional“ Investigación y Género: Sevilla, 3 y 4 de Julio de 2014. Sevilla: SIEMUS (Seminario Interdisciplinar de Estudios de las Mujeres de la Univesidad de Sevilla), 703–722. Available online at: https://idus.us.es/handle/11441/40954 (Accessed December 15, 2025).

[B14] Edelsztein V. C. (2023). Breaking down the gender pay gap through a machine learning model. Converg. Rev de Cienc Soc 30. doi: 10.29101/crcs.v30i0.20656

[B15] Ekamena Ntsama S. N. (2014). Gender wage gaps in Cameroon. Multidiscip. Rev. Employ. Unionism Work 9, 124–146.

[B16] Etoundi Atenga E. M. Chameni Nembua C. Meva Avoulou H. J. (2013). Wage Gaps Between Men and Women in Cameroon: Discrimination or Human Capital? A Subgroup Approach.

[B17] Franklin S. Walker C. Statistique Canada. (2010). Methodes et pratiques d'enquete. Statistique Canada. https://central.bac-lac.gc.ca/.item?id=12-587-x2003001-fra&op=pdf&app=Library

[B18] Fuller S. Kim Y. M. (2024). Women managers and the gender wage gap: workgroup gender composition matters. Work Occup. 51, 325–361. doi: 10.1177/07308884231178314

[B19] Georges-Kot S. (2020). Gender pay gaps: primarily the effect of working hours and occupation. Insee Prem. 1803, 1–4.

[B20] Giaimo R. Lo Magno G. L. (2014). A Distributional Approach for Measuring Wage Discrimination and Occupational Discrimination Separately. Cham: Springer, 251–261. doi: 10.1007/978-3-319-05552-7_22

[B21] Gouider A. (2009). “Determinants of women's activity in the Tunisian labor market and gender wage discrimination,” *Paper Presented at the GDRI DREEM Conference, Inequalities and Development in Mediterranean Countries*, Galatasaray University, Turkey, 21–23.

[B22] Houanti L. Dang R. Achabou M. A. Bruna M. G. (2022). Is there a wage gap between men and women in developing countries? A case study in Algeria. Quest. Manag. 1, 171–186. doi: 10.3917/qdm.218.0171

[B23] Johnson I. A. (2016). Analysis of the determinants of wage discrimination in Togo. CEDRES Stud. J. 5.

[B24] Mahugnon S. B. Hamdy B. G. (2023). Does the Beninese labor market discriminate? Afric. Sci. J. 3, 461–486.

[B25] Mincer J. Polachek S. (1974). Family investments in human capital: earnings of women. J. Polit. Econ. 82, S76–S108. doi: 10.1086/260293

[B26] Nazari S. (2022). Employment income gap in the Canadian labour market: intersection of gender, religion and visible minority status. J. Muslim. Minor. Aff. 42, 179–197. doi: 10.1080/13602004.2022.2113291

[B27] Oaxaca R. (1973). Male-female wage differentials in urban labor markets. Int. Eco. Rev. 14, 693–709. doi: 10.2307/2525981

[B28] OECD (2022). Understanding explained and unexplained differences between two groups through a counter-factual exercise: the Oaxaca- blinder decomposition. OECD Rural Stud. doi: 10.1787/c26abeb4-en

[B29] Polachek S. W. (1981). Occupational self-selection: a human capital approach to sex differences in occupational structure. Rev. Econ. Statist. 63, 60–69. doi: 10.2307/1924218

[B30] Programme d'Appui au Développement de l'Élevage (2021). Technical-Economic Reference on the Poultry Sector. Programme d'Appui au Développement de l'Élevage, 2021.

[B31] Putri Q. N. Firmadhani N. A. Shidiq F. A. Islami F. S. Panjawa J. L. (2025). “Dynamics of the gender wage gap in java island, 2024: an oaxaca–blinder analysis within the mincer human capital framework,” *The International Conference on Sustainable Economics Management and Accounting Proceeding*, (Vol 1), 2844–2852. doi: 10.32424/icsema.1.1.182

[B32] Sarma G. K. (2022). Exploring the gender pay gap: a systematic review of causes and magnitudes. Indian Sci. J. Res.Eng.Manage. 06, 1–9. doi: 10.55041/IJSREM11829

[B33] Sielska A. (2023). Introduction to Explaining the Gender Wage Gap. Edward Elgar Publishing, 1–3. doi: 10.4337/9781035312597.00005

[B34] Theodoropoulos N. Forth J. Bryson A. (2022). Are women doing it for themselves? Female managers and the gender wage gap^*^. Oxf. Bull. Econom. Stat. 84, 1329–1355. doi: 10.1111/obes.12509

[B35] Thiongane M. Kane A. (2023). Analysis of the gender wage gap in senegal: differences in productive skills or discrimination? World Dev. 51, 149–170.

[B36] UNDP A. (2016). Rapport sur le développement humain en Afrique 2016 Accélérer les progrés en faveur de l'égalité des genres et de l'autonomisation des femmes en Afrique. United Nations Development Programme (UNDP).

